# Fc-Elabela fusion protein attenuates lipopolysaccharide-induced kidney injury in mice

**DOI:** 10.1042/BSR20192397

**Published:** 2020-09-01

**Authors:** Feng Xu, Huifen Zhou, Man Wu, Hong Zhang, Yixian Zhang, Qingbin Zhao, Robert Brown, Da-Wei Gong, Lining Miao

**Affiliations:** 1 Department of Nephrology, The Second Hospital of Jilin University, Changchun, China; 2 Division of Endocrinology, Diabetes and Nutrition, Department of Medicine, University of Maryland School of Medicine at Baltimore, Baltimore, Maryland, USA

**Keywords:** acute kidney injury (AKI), Elabela (ELA), fusion protein, liposaccharide (LPS)

## Abstract

Endotoxemia-induced acute kidney injury (AKI) is a common clinical condition that lacks effective treatments. Elabela (ELA) is a recently discovered kidney peptide hormone, encoded by the gene *apela*, and has been reported to improve cardio-renal outcomes in sepsis. However, ELA is a small peptide and is largely unsuitable for clinical use because of its short *in vivo* half-life. In the present study, we evaluated the potential renoprotective effects of a long-acting constant fragment (Fc)-ELA fusion protein in liposaccharide (LPS)-induced AKI in mice. LPS administration in mice for 5 days greatly lowered the gene expression of *apela* and impaired kidney function, as evidenced by elevated serum creatinine and the ratio of urine protein to creatinine*.* In addition, renal inflammation and macrophage infiltration were apparent in LPS-challenged mice. Treatment with the Fc-ELA fusion protein partially restored *apela* expression and attenuated the kidney inflammation. Moreover, LPS treatment induced reactive oxygen species (ROS) production and apoptosis in kidney HK-2 cells as well as in the mouse kidney, which were mitigated by ELA or Fc-ELA treatment. Finally, we found that ELA promoted the survival of HK-2 cells treated with LPS, and this action was abolished by LY204002, a PI3K/Akt inhibitor. Collectively, we have demonstrated that the Fc-ELA fusion protein has significant renoprotective activities against LPS-induced AKI in mice.

## Introduction

Sepsis is a common severe clinical condition, often resulting in multiple organ injury and leading to significant mortality [1,2]. Sepsis-induced acute kidney injury (AKI) is a leading cause of deaths in hospitalized patients with mortality rate of up to 48% [3,4]. Currently, no effective pharmacological interventions for septic AKI are available [5] and thus, there is an unmet urgent need for development of novel therapeutics to treat this severe medical complication.

Liposaccharide (LPS)-induced AKI is a commonly used animal model for the study of sepsis-associated kidney injury. It is well-established that LPS activates the innate immune system through pattern recognition receptors such as toll-like receptors (TLRs), leading to a cascade of inflammatory responses, physiological perturbations and tissue damage [6]. For example, the interaction of LPS with TLRs activate tubular cells, as well as interstitial macrophages or dendritic cells, in turn stimulating local production of cytokines and chemokines in the kidney [7]. Inflammatory responses will also lead to increased production of nitric oxide (NO) and reactive oxygen species (ROS) and manifest as inflammatory cell infiltration, tissue destruction and cell death via necrosis and apoptosis [8,9]. Renal dysfunction [1,10,11] ensues as a result.

The apelinergic system is composed of the apelin receptor (APJ) with two endogenous peptide ligands/hormones: apelin and the newly discovered Elabela (ELA, Apela or Toddler) [12,13] and is emerging as a protective system against tissue damage in ischemia and inflammation [14]. Many studies have shown that apelin can attenuate multiple organ injuries following hemorrhagic shock [15] and in sepsis [16–18]. We have previously reported that constant fragment (Fc)-apelin, a long-acting form of apelin, can ameliorate LPS-induced liver damage in mice [19]. Likewise, ELA has recently been reported to be protective against sepsis-induced organ damage [18]. However, whether ELA is renoprotective against sepsis-induced AKI is yet to be evaluated.

ELA is a peptide of 32 amino acids and is presumed to be cleaved to peptide fragments of ELA-21, ELA-14 and ELA-11 [12–14]. Therapeutic peptides of less than 50 amino acids are usually unsuitable for clinical use because of short *in vivo* half-life [20]. To extend ELA’s half-life, we fused the Fc domain of the human immunoglobulin IgG to ELA-21, resulting in Fc-ELA-21 (referred to hereafter as Fc-ELA unless otherwise specified) which has an extended plasma half-life of ∼44 h (vs.∼13 min of the native peptide [21]) and is biologically active. Administration of Fc-ELA successfully mitigated cardiac dysfunction in a model of myocardial infarction [21]. We hypothesized that the long-acting Fc-ELA, which is clinically applicable, might also mitigate sepsis-induced kidney inflammation and injury. We thus tested this hypothesis by investigating the effects of Fc-ELA on LPS-induced AKI in mice.

## Materials and methods

### Reagents

LPS from *Salmonella enterica* was purchased from Sigma–Aldrich (St. Louis, MO, U.S.A.), LY294002 from Cell Signaling Technology (Danvers, MA) and dihydroethidium (DHE) from Invitrogen (Eugene, OR). ELA-21 peptide was made by GenScript (Piscataway, NJ) and Fc-ELA was made by conjugating the human IgG Fc fragment with ELA-21 at the N-terminus as previously described [21].

### Animal studies

The animal protocols were approved by the Institutional Animal Care and Use Committee of the University of Maryland School of Medicine and the animal studies were performed at the University of Maryland School of Medicine. C57/BL6 female mice, at 6 weeks old, were obtained from Charles River (Wilmington, MA) and used after 1 week of quarantine and acclimatization. Experimental animals were randomly divided into four groups: (1) The control group (CON) received PBS (vehicle of LPS) intraperitoneally (i.p.) and subcutaneously (s.c.); (2) The Fc-ELA group was treated with Fc-ELA (1 mg/kg/day), s.c. and PBS, i.p.; (3) The LPS group was challenged with LPS (1 mg/kg) i.p. and PBS, s.c.; and (4); The LPS/Fc-ELA group received LPS (1 mg/kg), i.p. and Fc-ELA (1 mg/kg), s.c. Mice were administered with vehicle, LPS and/or Fc-ELA daily for five consecutive days. Six hours after final injection, the mice were anesthetized by CO_2_ gas and killed by cervical dislocation. Blood samples were collected and serum samples were separated and stored at −20°C until analysis. Kidney specimens were embedded in the optimal cutting temperature compound Tissue-Tek (Sakura, CA), fixed in 10% neutral buffered formalin for histological analysis or snap-frozen in liquid nitrogen.

### Quantitative real-time PCR

Total RNAs were prepared from the snap-frozen tissue specimens using TRIzol (Invitrogen), and reverse transcription was carried out in a reaction containing 1 μg of total RNA, poly(dT) primer, and Moloney murine leukemia virus reverse transcriptase using the Transcriptor First Strand cDNA Synthesis kit (Promega). Quantitative real-time PCR (qRT-PCR) was conducted on a LightCycler480 using the LightCycler 480 SYBR Green I Master Mix (Roche Diagnostics). The amplification protocol was as follows: 95ºC/5 min, then (95ºC/10 s, 60ºC/20 s, and 72ºC/30 s) × 45 cycles. Following amplification, a dissociation curve analysis was performed to ensure purity of the PCR product. qPCR primers were 5′-tgcattccacttcattctcg-3′ (forward) and 5′-gttgccatcctgaggttgtt-3′ (reverse) for apela, 5′-actatggggctgacaaccag-3′ (forward) and 5′-ggcaaagtcaccacaaaggt-3′ (reverse) for APJ, 5′-gaggaaatttcgcagacagc-3′ (forward) and 5′-gaggaacttggtgggtgaga for apelin (reverse), and 5′-tggaccttccaggatgaggaca-3′ (forward) and 5′-gttcatctcggagcctgtagtg-3′ (reverse) for IL-1β. Primers for TNFα and IL-6 were described previously [14]. β-actin mRNA was used for normalization of cDNA loading as an internal control. The relative expression of the target genes was determined by the 2^−ΔΔ*C*_T_^ method [22].

### Protein and creatinine assays

At the end of the animal experiment, serum and urine were collected for total protein and creatinine measurements. Total protein concentrations were determined using the Pierce BCA protein assay kit (Thermo Scientific, Rockford, IL, U.S.A.) according to manufacturer’s protocol.

Urine was collected by micturition on a Petri dish and subsequent aspiration with a 200-μl pipette [23]. In brief, pressure was applied in a massaging fashion at both sides of the lower back near the tail with the thumb on one side and the fore and middle fingers on the other side of a mouse, rubbing up and down of the caudal area of the back of the mouse to facilitate urination. Once the mouse started urinating, the experimenter gently pulled the skin at the lower back upward with the massaging fingers to immobilize the mouse and to prevent premature halt of urination. The voided urine was aspirated using a 200-μl pipette. Creatinine was measured by using a creatinine assay kit (Crystal Chem Inc., IL, U.S.A.).

### Cell studies

The immortalized human proximal tubular epithelial cell lines HK-2 (ATCC CRL-2190, Manassas, VA) were grown in Dulbecco’s modified Eagle’s medium (DMEM) supplemented with 10% FBS, 2 mM l-glutamine, 100 units/ml penicillin, and 100 μg/ml streptomycin (complete medium) at 37°C. For apoptosis studies, cells were plated and grown on cover slips in a well of a six-well plate in complete medium to approximately 80% confluence and then changed to serum-free medium with ELA-21 (2 μM) and/or LPS (2 μg/ml) for 12 h before fixation with 4% paraformaldehyde for 30 min.

### Immunostaining and histology studies

Fixed kidney tissues were processed routinely, embedded in paraffin, cut into 5-μm thickness slices and stained with Hematoxylin and Eosin (H/E). For immunohistochemistry (IHC), the paraffin-embedded sections were subjected to deparaffination, 3% H_2_O_2_ treatment and antigen retrieval, and then sequential incubation with the primary antibody F4/80 (BM8, eBioscience) and HRP-conjugated goat anti-rabbit IgG (KPL, Gaithersburg, MD), as described previously [19]. Kidney tissue apoptosis was assessed by terminal deoxynucleotidyl transferase-mediated deoxyuridintriphosphate nick-end labeling (TUNEL) on tissue sections or HK2 cells grown on cover slides. The TUNEL staining and Methyl Green counterstaining were performed using DeadEnd™ Fluorometric TUNEL System (Promega, Madison, WI, U.S.A.), according to the manufacturer’s protocol. Fluorescein-labeled TUNEL positive cells were counted under ×400 magnification with a microscope (Olympus IX-51). The apoptotic index was calculated as the percentage of TUNEL-positive cells/total number of cells.

### DHE staining

The production of superoxide in oxidative stress were measured in the frozen kidney tissue sections and HK-2 cell cultures using the oxidative fluorescent dye DHE. Kidney cryosections at 5-μm thickness were stained with the superoxide-sensitive dye DHE (1 μmol/l) in a light-protected and humidified chamber for 30 min at 37°C. Three nonoverlapping images per sample were averaged with a fluorescence microscope (Olympus IX-51) and the signal was quantified by Image-Pro Plus 6.0 (Bethesda, MD, U.S.A.).

### Western blotting

Kidney tissues were homogenized and HK-2 cells were sonicated in RIPA buffer (TekNova, CA, U.S.A.). After centrifugation, supernatants were dissolved in Laemmli Buffer. The dissolved proteins were separated by sodium dodecyl sulfate/polyacrylamide gel electrophoresis (SDS/PAGE) and transferred to polyvinylidene difluoride membranes (Millipore, MA, U.S.A.). The membranes were blocked with 5% nonfat milk for 1 h and then were incubated overnight at 4°C with the following primary antibodies: Bcl-2, Bax (Cell Signaling, Danvers, MA, U.S.A.), ELA (AbboMax, San Jose, CA), or GAPDH (Proteintech, Chicago, IL, U.S.A.). After washing, membranes were incubated with the appropriate secondary antibodies and developed by enhanced chemiluminescence (Luminata, Millipore, Billerica, MA, U.S.A.). Protein bands were imaged and quantified by using Alpha View-Fluor ChemQ software (Alpha Innotech Gel Imaging System, San Jose, CA).

### MTT assay

Cell viability and proliferation were measured by using 3-(4,5-dimethyl-2-thiazolyl)-2,5-diphenyl tetrazolium bromide (MTT) (Acros Organic, Geel, Belgium) according to the manufacturer’s protocol. In brief, cells were seeded at 8000 cells per well into 96-well plates. At 80% confluence, cells were pretreated with Fc-ELA (2 μM) and/or LY294002, then incubated with LPS (2 μg/ml) for 12 h, followed by incubation with MTT (5 mg/ml) for an additional 4 h. The absorbance at the 490-nm wavelength was measured using a microplate reader after medium removal and the addition of 100 μl DMSO. The control group consisting of untreated cells was considered to be 100% viable. Results are expressed as the percentage of viable cells when compared with the control groups.

### Data analysis

All data are presented as mean ± SEM. Statistical analyses were performed with one-way ANOVA followed by post-hoc Bonferroni’s test. Differences were considered statistically significant at the level of *P*<0.05.

## Results

### Expression of *apela* is decreased in LPS-treated mice

**Figure 1:**
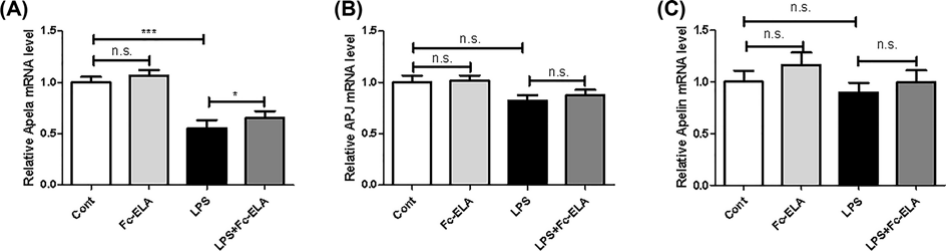
Effect of Fc-ELA on kidney expression of *Apela, APJ*, and *apelin* in LPS-treated mice qPCR was conducted for *apela* (**A**), *APJ* (**B**), and *apelin* (**C**) in the kidney of mice receiving PBS (Cont) or Fc-ELA (1 mg/kg) with or without LPS (1 mg/kg) for 5 days. Data are expressed as mean + SE (*n*=8). *P<0.05, ***P<0.001 and n.s., no statistical significance.

The *apela* gene, encoding ELA, is known to be selectively expressed in the kidney. To assess whether the expression of *apela* was affected by LPS and/or exogenous ELA administration, we measured *apela* mRNA expression in the kidney of the mice receiving LPS and/or Fc-ELA treatment. As shown in Figure 1A, Fc-ELA treatment increased *apela* expression slightly, but not significantly in the control group. However, *Apela* expression was significantly decreased by 44.8% (*P*<0.001) after LPS challenge and co-treatment with Fc-ELA restored its expression level by 18.1% (*P*<0.05). On the other hand, no significant changes in *APJ* or *apelin* expression was noted in any of the treatment groups (Figure 1B,C).

### Fc-ELA alleviates kidney injury in LPS-treated mice

**Figure 2:**
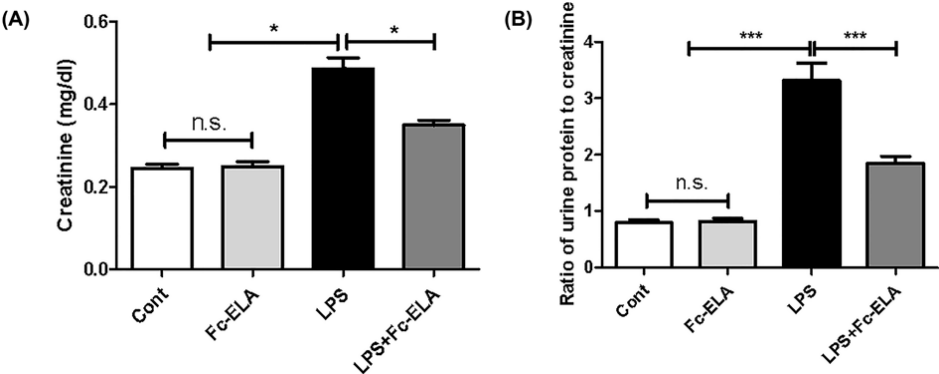
Effect of Fc-ELA on serum creatinine and the ratio of urine protein to creatinine in different experimental groups (**A**) Effect on serum creatinine. (**B**) Effect on the ratio of urine protein to creatinine. Mice were administered PBS (Cont) or Fc-ELA (1 mg/kg) with or without LPS (1 mg/kg) daily for 5 days. Serum and urine sample were collected in different experimental groups. Data are expressed as mean + SE (*n*=9). *P<0.05, ***P<0.001 and n.s., no statistical significance.

We then assessed kidney function by measuring serum creatinine and the urine protein to creatinine ratio, two widely used indicators of renal function [24]. As shown in Figure 2A, compared with the control group, LPS administration significantly elevated serum creatinine levels from 0.24 to 0.48 mg/dl (*P*<0.001). Co-treatment with Fc-ELA significantly attenuated the LPS-induced increase to only 0.35 mg/dl (*P*<0.05), though the creatinine levels remained higher than the control group. Fc-ELA alone had no effect on serum creatinine in mice. Furthermore, LPS administration significantly increased the ratio of urine protein to creatinine (*P*<0.001), while co-treatment with Fc-ELA significantly attenuated the ratio of protein to creatinine in LPS-challenged mice (*P*<0.05; Figure 2B). The present study demonstrated that LPS-induced impairment of kidney function was partially reversed by Fc-ELA co-administration.

### Fc-ELA attenuates kidney injury and inflammatory response in LPS-treated mice

**Figure 3:**
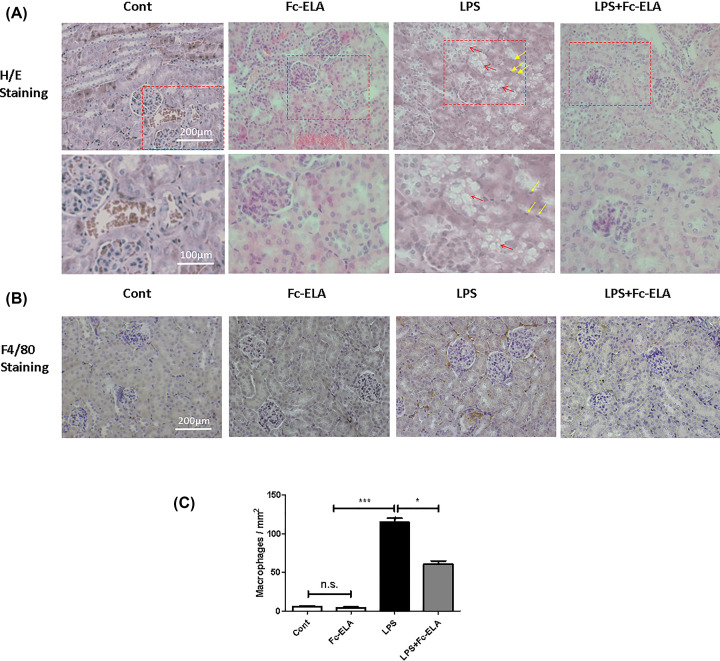
Fc-ELA treatment ameliorates LPS-induced kidney damage and macrophage infiltration (**A**) Representative H/E staining images of kidney tissue sections. Red arrows indicate vacuolation and yellow arrows indicate nuclear pyknosis in the LPS group. (**B**) Representative tissue section images of macrophage marker F4/80 staining and quantification (**C**). Data are expressed as mean + SE (*n*=5). *P<0.05, ***P<0.001 and n.s., no statistical significance.

We next conducted histological studies to examine the effect of Fc-ELA on the murine kidney. Renal morphology appeared normal in H/E staining in the mice receiving PBS and Fc-ELA. However, in the LPS-treated mice, renal tubular structure appeared vacuolar with some tubular epithelial cells appearing to show nuclear pyknosis (Figure 3A), a finding significantly attenuated by co-treatment of Fc-ELA. The degree of macrophage infiltration, an indicator of the inflammatory response, was quantified by the staining of F4/80, a macrophage-specific marker [25,26]. As shown in Figure 3B,C, LPS remarkably increased the F4/80 labeling, but this effect was substantially blunted by Fc-ELA treatment. Nevertheless, the extent of macrophage staining in the LPS + Fc-Ela group remained higher than the PBS or Fc-ELA-treated group. These data indicated that the Fc-ELA fusion protein had a renoprotective and anti-inflammatory effect.

**Figure 4:**
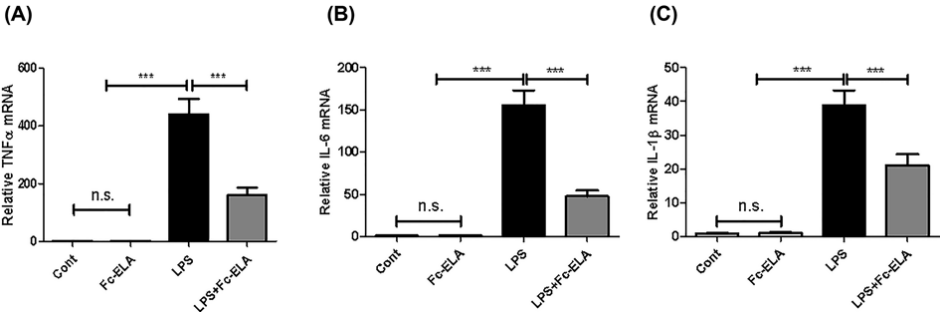
Effect of Fc-ELA on renal expression of TNFα and IL-6 genes Quantitative PCR (qPCR) of proinflammatory cytokines TNFα (**A**), IL-6 (**B**), and IL-1β (**C**). Gene expression is normalized with β-actin. Data are expressed as mean + SE (*n*=5). ***P<0.001 and n.s., no statistical significance.

Given the pronounced increase in kidney macrophages of mice receiving LPS and Fc-ELA treatment, we measured mRNA expression of the inflammatory cytokines TNFα, IL-6, and IL-1β. As shown in Figure 4, compared with the PBS or Fc-ELA controls, the TNFα, IL-6, and IL-1β. Transcript levels were significantly elevated after the administration of LPS, and the degree of elevation was attenuated by approximately 63.27, 69.31, and 45.5%, respectively, with Fc-ELA co-administration.

### ELA reduces LPS-induced ROS production in kidneys and HK-2 cells

**Figure 5:**
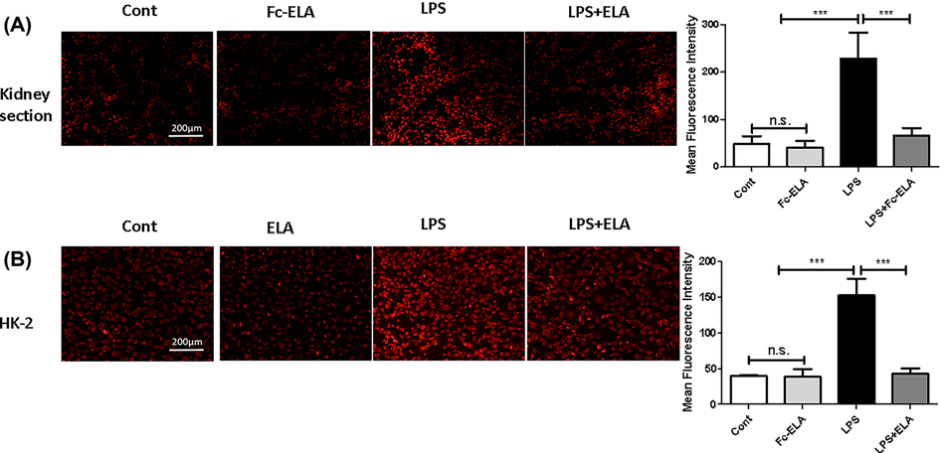
Detection of ROS in kidneys and HK-2 cells by DHE staining (**A**) Kidney tissue sections of mice receiving PBS, Fc-ELA (1 mg/kg) and/or LPS were stained with DHE and the fluorescence intensity was quantified by fluorescence microscopy. Quantification of ROS production was done by measuring fluorescence intensity. (**B**) HK-2 cells were pre-treated with PBS/albumin (Cont) or Fc-ELA (0, 5 μM) for 30 min and then with or without LPS (2 μg/ml) for 1 h. The cells were incubated with DHE for 20 min and quantified by microscopy. Quantification of ROS production was done by measuring fluorescence intensity. Data are expressed as mean + SE (*n*=9). ***P<0.001 and n.s., no statistical significance.

ROS overproduction is a hallmark of inflammation. To determine whether ROS were implicated in ELA’s anti-inflammatory activity [27], we measured ROS levels in kidney tissue sections and in HK-2 cells by DHE fluorescence (Figure 5). Compared with the control group, mice injected with LPS showed a significant increase (*P*<0.01) in kidney DHE staining. Co-treatment with Fc-ELA markedly lowered the DHE intensity (Figure 5A). In contrast, PBS or Fc-ELA alone had no effect on ROS production. In the human kidney cell line HK-2, ROS production was also induced by LPS and ameliorated by ELA-21 co-treatment (Figure 5B). Thus, ELA suppressed LPS-induced ROS production both *in vivo* and *in vitro*.

### ELA protects against LPS-induced apoptosis in the murine kidney and in HK-2 cells

**Figure 6:**
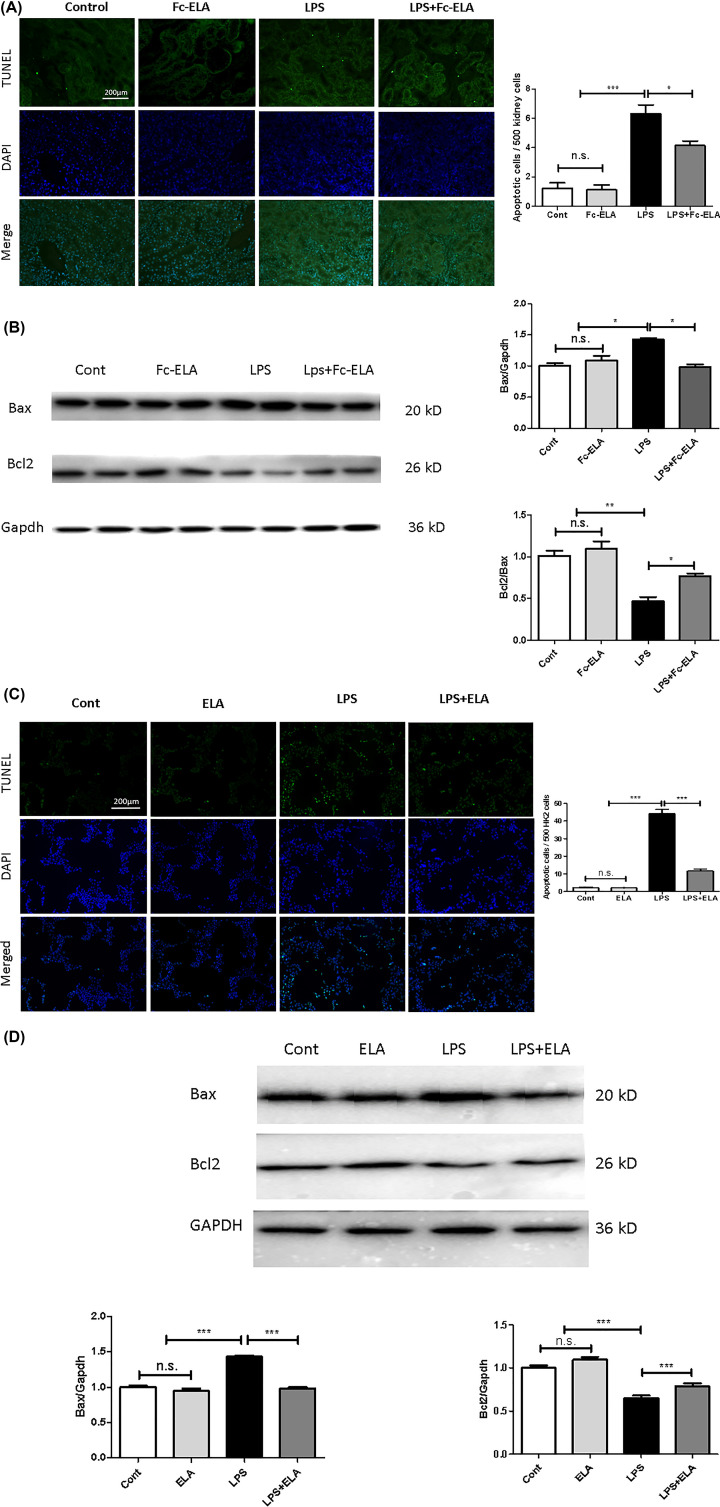
ELA suppresses LPS-induced apoptosis in kidneys and HK-2 cells Apoptosis analyses of kidney tissues. (**A**) Upper panels, representative TUNEL staining images (green color); lower panels, DAPI staining (blue color). (**B**) Representative Western blots of the apoptosis related proteins Bax and Bcl2, in HK-2 cells. (**C**) Upper panels, representative TUNEL staining images (green color); lower panels, DAPI staining (blue color). (**D**) Western blot analyses. Data are expressed as mean + SE (*n*=5 for A,C,D; *n*=6 for B). *P<0.05, **P<0.01, ***P<0.001, and n.s., no statistical significance.

As nuclear pyknosis (Figure 3A) is a sign of cell death, which could be due to apoptosis, we next quantified apoptotic cells by performing a TUNEL assay in kidney tissue slides. As depicted in Figure 6A, very few apoptotic cells were seen in the control or Fc-ELA-treated animals. Remarkably, LPS administration increased the number of apoptotic cells by five-fold, most of which appeared to be in tubular cells. Co-treatment with Fc-ELA significantly reduced the number of apoptotic cells. We next measured the apoptotic proteins Bcl-2 and Bax by immunoblotting. Figure 6B showed that Bax expression was increased significantly, whereas Bcl2 expression was decreased in LPS-challenged kidneys compared with the expression levels in untreated mice. Notably, Fc-ELA co-treatment antagonized the LPS effect by decreasing Bax expression and increasing Bcl2 expression (Figure 6B). We further showed whether or not ELA’s anti-apoptotic action was direct in HK-2 cells by TUNEL assay (Figure 6C). Bax expression was significantly elevated in LPS-treated HK-2 cells, whereas ELA-21 peptide pretreatment decreased Bax expression. Bcl-2 expression was significantly decrease in LPS treated HK-2 cells, whereas ELA-21 peptide pretreatment increased the Bcl2 expression (Figure 6D). Thus, ELA was protective against LPS-induced renal apoptosis both *in vitro* and *in vivo*.

### Implication of the PI3K/Akt signaling pathway in ELA-mediated cell survival

**Figure 7:**
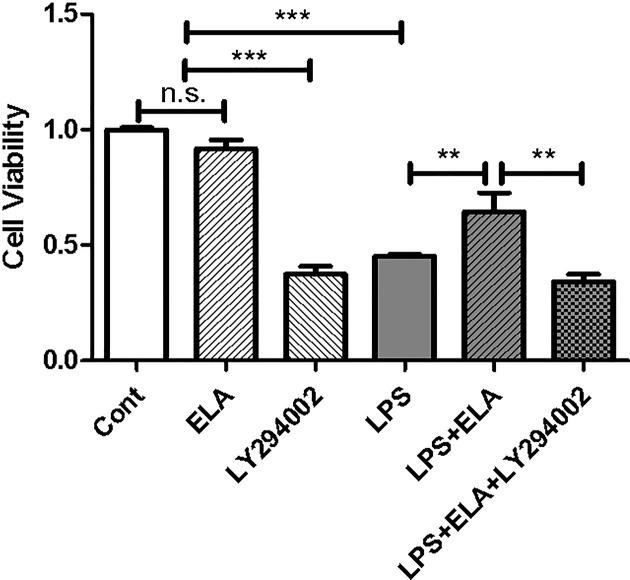
Protection of LPS-induced cell death by ELA A cell viability assay was conducted in HK-2 cells treated with LPS, Fc-ELA and/or the PI3K/Akt inhibitor LY294002 and measured by MTT. Data are expressed as mean + SE (*n*=5). **P<0.01, ***P<0.001 and n.s., no statistical significance.

Activation of the PI3K/Akt signaling pathway is known to be critical for cell survival [28] and ELA is a potent activator of the pathway [29]. Thus, we assessed cell viability in response to LPS and/or ELA in the presence or absence of the PI3K inhibitor LY294002. As shown in Figure 7, LPS treatment reduced cell viability by 54.7% (*P*<0.001), and co-treatment with ELA partially restored the viability to 64.4% of the control (*P*<0.01), an effect blocked by LY294002 treatment, indicating that the PI3K/Akt signaling is involved in ELA-mediated cytoprotection.

## Discussion

ELA is a newly discovered endogenous ligand of APJ and is selectively expressed in the kidney, though its local and systemic functions remain to be fully understood. Recently, Conquerl et al. [18] have reported that, in a rat model of CLP (cecal ligation puncture)-induced sepsis, continuous infusion of ELA alleviates cardio-renal dysfunction by improving cardiac hemodynamics and urinary output. Notably, ELA appears to be more effective than apelin-13 in its protective effects [18]. However, the ELA peptide has a very short *in vivo* half-life and is not applicable clinically without modification. Our group has produced Fc-ELA-32 and Fc-ELA-21 fusion proteins and found that Fc-ELA-32 is cleaved whereas Fc-ELA-21 remains intact during *in vitro* production. Further studies show that Fc-ELA-21 (Fc-ELA) has an *in vivo* half-life of ∼44 h and can significantly improve heart function in a model of myocardial infarction [21] with daily administration. Since ELA appears to exert an organ protective effect in sepsis [18], we investigated the renoprotective effects of Fc-ELA in LPS-induced endotoxemia.

LPS-induced kidney injury is known to cause renal dysfunction [30] associated with pathological changes through a complex mechanism involving overproduction of ROS [31,32], macrophage infiltration and inflammation, and cell death by apoptosis [1,33]. In this study, mice receiving LPS administration for 5 days developed renal dysfunction as demonstrated by increased levels of serum creatinine and the urine albumin/creatinine ratio, which is associated with inflammation and structural damage to the kidney. Concomitant treatment of Fc-ELA significantly improved kidney function and reduced tissue damage. At the histological level, LPS-treated kidneys showed increased macrophage infiltration, ROS overproduction and apoptosis, all of which were reduced by Fc-ELA co-treatment in the mice. Our *in vitro* experiments revealed that ELA was capable of suppressing LPS-induced ROS production [27] and apoptosis, and promoting cell survival in human kidney HK-2 cells. These observations are consistent with publications wherein exogenous administration of apelin or ELA can decrease inflammation *in vitro* and *in vivo* [14,34] and promote cell survival [19,35,36].

It is interesting to note that *apela* (the elabela gene) expression was reduced by LPS, but *APJ* was not in LPS-treated mice. Although both *apela* and *APJ* are highly expressed in the kidney, their expression patterns are different; *apela* is mostly localized to tubular structures whereas *APJ* and *apelin* are more widely distributed [37–39]. LPS appeared to selectively cause apoptosis in the tubular cells (Figure 6A), which may explain the significantly decrease in whole tissue *apela*, but not APJ and *apelin*. Our finding that *apela* expression is decreased by LPS and partially restored by Fc-ELA suggests that ELA levels may be a marker of kidney function, which is in line with recent reports that circulatory ELA levels correlate with the ratio of albumin to creatinine in patients with diabetic nephropathy [40]. In light of the high tissue expression of *apela* in the kidney, ELA may play a protective role locally against injuries by, e.g. inflammation and ischemia [14], which warrants further investigation.

Overall, our work has shown Fc-ELA’s comprehensive renoprotective effects against LPS-induced kidney injury. Given Fc-ELA’s prolonged *in vivo* half-life, it may be a therapeutic candidate for septic kidney injuries.

## Abbreviations

AKI: acute kidney injury
APJ: apelin receptor
Bax: Bcl2 associated X
Bcl2: B-cell lymphoma 2
DHE: dihydroethidium
ELA: Elabela
Fc: constant fragment
GAPDH: glyceraldehyde-3-phosphate dehydrogenase
H/E: Hematoxylin and Eosin
HRP: horseradish peroxidase
IL: interleukin
i.p.: intraperitoneally
LPS: liposaccharide
MTT: 3-(4,5-dimethyl-2-thiazolyl)-2,5-diphenyl tetrazolium bromide
ROS: reactive oxygen species
s.c.: subcutaneously
TLR: toll-like receptor
TUNEL: terminal deoxynucleotidyl transferase-mediated deoxyuridintriphosphate nick-end labeling
